# Management of Crown-Root Fracture in Primary Canine by Surgical Extrusion: A Case Report with 1-Year Follow-Up

**DOI:** 10.1155/2018/3753807

**Published:** 2018-08-19

**Authors:** I. Kanimozhi, Mahesh Ramakrishnan, Dhanalakshmi Ravikumar, Ningthoujam Sharna

**Affiliations:** ^1^Government Medical College, Tuticorin, Tamil Nadu, India; ^2^Department of Pedodontics and Preventive Dentistry, Saveetha Dental College, Chennai, Tamil Nadu, India; ^3^Private Pratice, Imphal, Manipur, India

## Abstract

Complicated crown-root fractures of primary teeth often present with a greater challenge to the pediatric dentist. Extraction of the involved tooth is the routine treatment indicated. But, early loss of this primary tooth may lead to esthetic and psychological problems and also causes a detrimental effect on the development of occlusion and the alveolar bone. The present case report described the management of crown-root fracture in a primary canine by surgical extrusion and showed a satisfactory prognosis at one-year follow-up.

## 1. Introduction

Trauma to the teeth and orofacial region is most commonly encountered in children compared to adults. The child age and behaviour management play an important role in deciding the treatment protocol. The fracture line originates in the crown portion of the tooth, extends apically into the root in an oblique direction, and frequently exposes the pulp which is termed as a complicated crown fracture. The management protocol should consider both the function and the esthetics of the fractured tooth [1]. Various treatment approaches are indicated depending on the tooth fracture, the age of the child, and the location of the degree of level of fracture. When the fracture line extends below the gingival margin, there is a high chance of microleakage from the gingival crevicular fluid and difficulty in isolation for postendodontic restorations. In such cases, extraction of the fractured tooth is often indicated [1]. When the fracture involves the anterior teeth, it can lead to esthetic and psychological problems, but also bring detrimental effect on the development of occlusion and the alveolar bone [2]. Therefore, prevention of injury and conservation of severely traumatised teeth are eminently significant whenever possible [3].

Surgical tooth extrusion is considered to be one of the most favourable treatment options when the fracture line extends subgingivally. The procedure involves severing the bone root periodontal attachment utilising a surgical instrument to place the root in a more coronal position [1–4]. The extruded tooth is then stabilised using a semirigid splint for a maximum of 3 weeks for ideal periodontal healing. Surgical extrusion is a one-step procedure which is biologically acceptable and less time to consume than orthodontic extrusion in the management of horizontal and oblique root fracture [1–3]. Literature review identified case reports of successful management involving surgical extrusion in permanent teeth. But in cases involving primary teeth, the only treatment opinion available is extraction followed by space management.

The present article describes a case of management of crown-root fracture in a primary canine by surgical extrusion, when followed up for 12-month duration that showed a satisfactory prognosis.

## 2. Case Report

**Figure 1:**
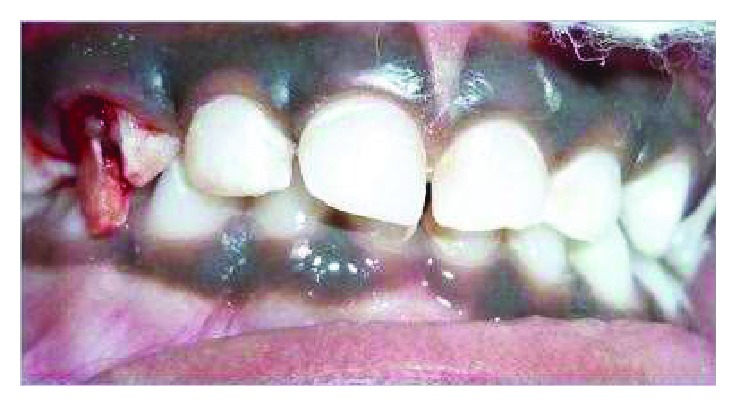
Preoperative photograph depicts fractured 53.

**Figure 2:**
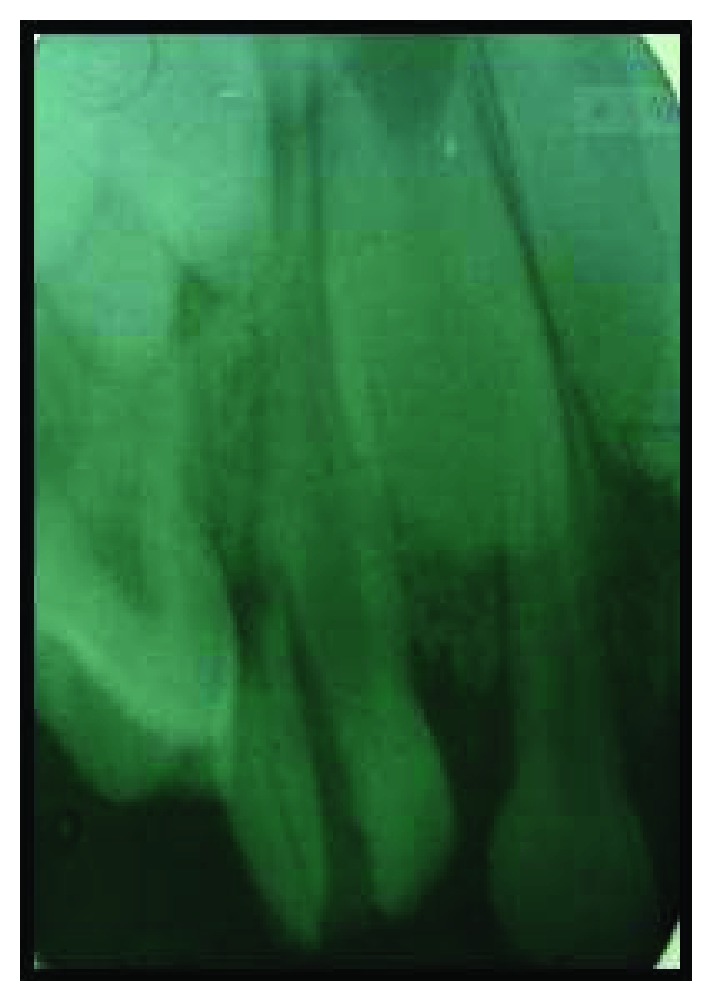
Preoperative radiograph depicts oblique crown-root fracture extending beyond cementoenamel junction.

A 6-year-old girl presented to the Department of Pediatric and Preventive Dentistry with the history of trauma in her right upper front region of the jaw. She had a fall on the school ground while playing 1 hr before the presentation, had no symptoms of nausea, and did not have any discharge or bleeding from her nose. The medical history was unremarkable and did not have any history of daily medication. No gross facial asymmetry was evident. She had no abrasions on the lower or upper lip. Her temporomandibular joints were functioning within limits and with no clicking, pain, or any abnormal mandibular deviation. On intraoral examination, the patient had a complete set of primary dentition and had moderate oral hygiene, with mild plaque deposits at the gingival margins. There was a fracture in the right maxillary canine (tooth number 53) with the oblique fracture line extending subgingivally (Figure 1). Radiograph (IOPA) taken in the right anterior region of the jaw (tooth number 53) revealed evidence of fracture line running 2 mm below the cementoenamel junction and involving the pulp (Figure 2).

**Figure 3:**
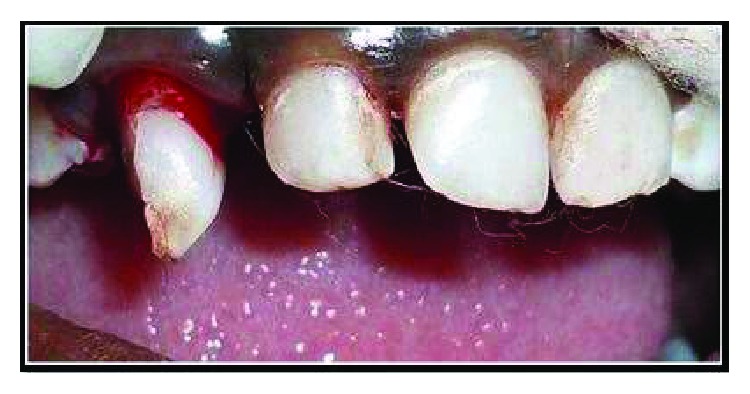
Surgically extruded 53.

**Figure 4:**
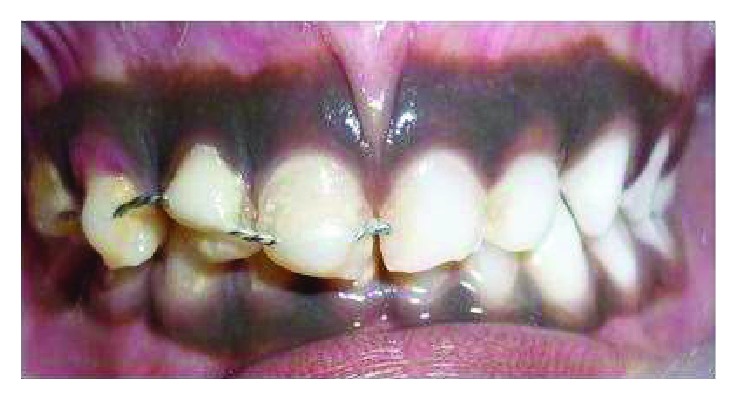
Surgically extruded 53 stabilised using composite splint.

**Figure 5:**
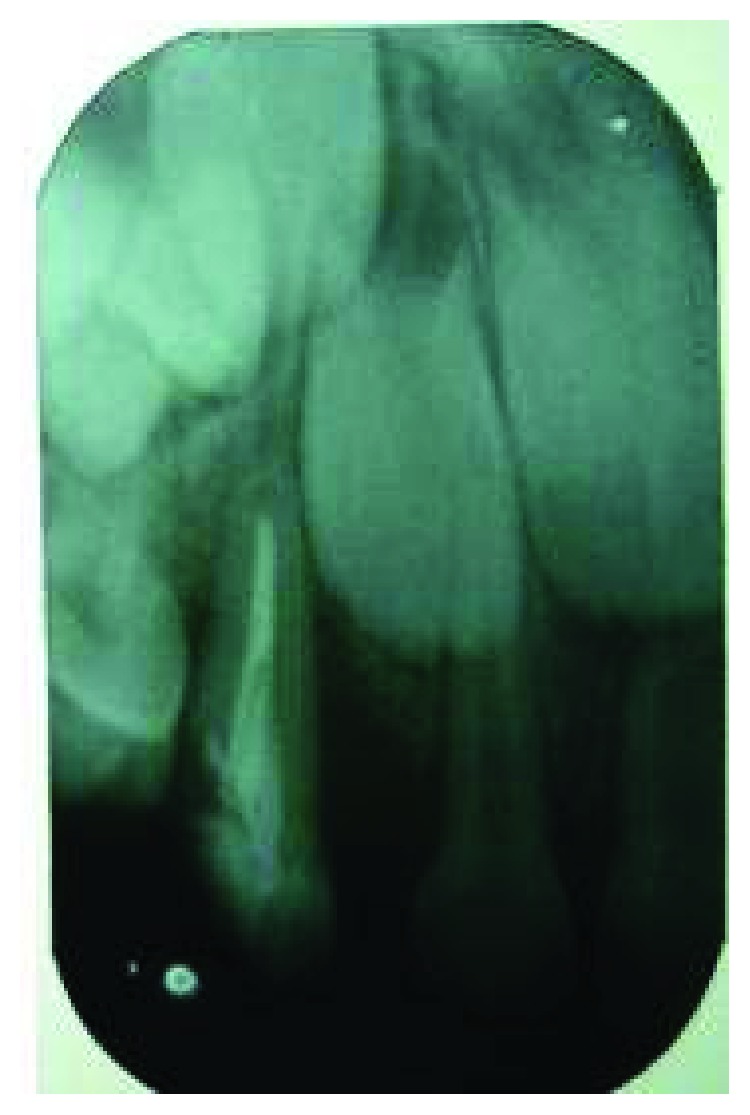
Radiograph taken at 6th-month follow-up.

**Figure 6:**
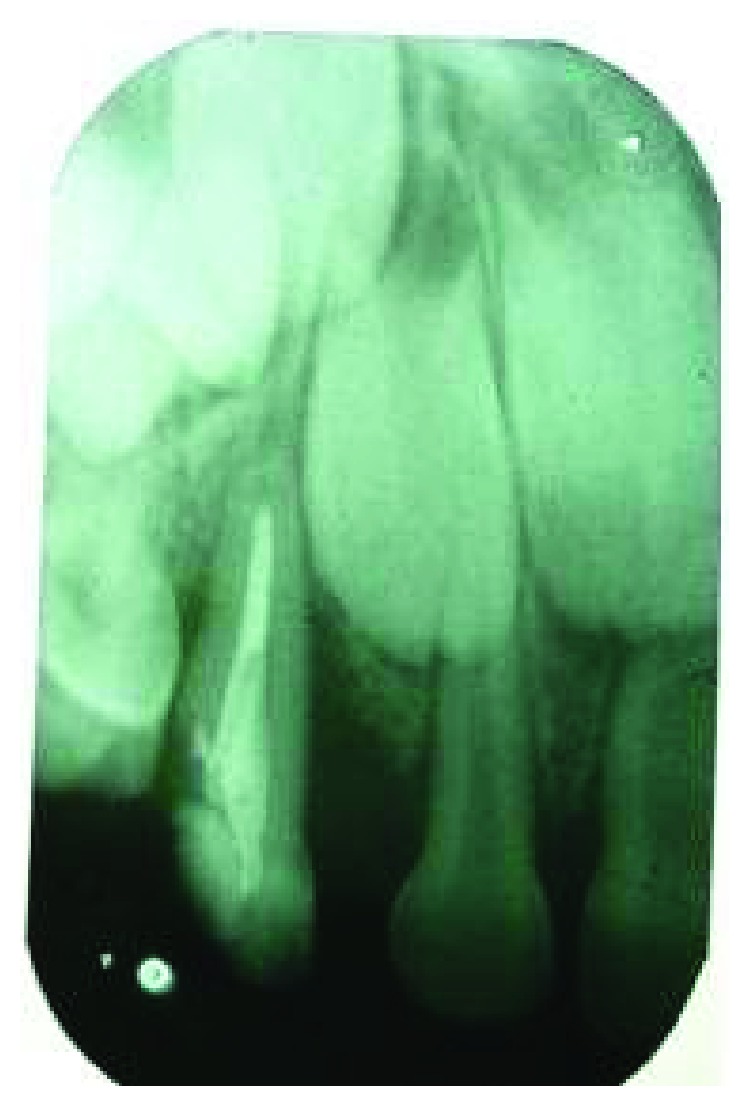
Radiograph taken at 12th-month follow-up.

The treatment options and prognosis for tooth 53 were discussed with the patient's mother. Preoperative photographs were taken, and local anaesthesia was administered (2% xylocaine with 1 : 80,000 adrenaline) on the buccal and palatal aspects of tooth 53. The mobile tooth fragment was extracted, and the remaining tooth structure was surgically extruded using a maxillary anterior forceps (Figure 3). Occlusion was checked to ensure that there are no occlusal interferences. Acid etching of tooth numbers 51, 52, 53, and 54 was done. The bonding agent was applied, and a semirigid splint was placed and stabilised using a flowable composite (Figure 4). The patient was advised to consume a soft diet and to be meticulous with her oral hygiene. A 0.2% chlorhexidine gluconate mouthwash was also prescribed twice daily for the next two weeks. The patient was reviewed a day following her initial presentation, and pulpectomy was carried out on the next day and obturated using Metapex. Composite splinting was removed at the end of the third week, and polishing was done. Mobility was checked, and radiographs were taken. On eight weeks following trauma, a periapical radiograph and photograph were updated. The patient was followed up periodically at the 3rd month, 6th month, and 12th month (Figures 5 and 6).

## 3. Discussion

**C**rown-root fracture typically presents as a fracture line that originates in the crown portion of the tooth which extends apically in an oblique direction frequently with pulp exposure. The treatment protocol for primary teeth with crown-root fracture as recommended by IADT guidelines is to leave the tooth untreated if the coronal fragment is not displaced or extracting the coronal segment with repositioning and splinting might be considered [5]. In severe cases, when there is crown-root fracture which is extending into the subgingiva involving the primary teeth, it is indicated for extraction. As a result of this protocol, many root-fractured primary teeth were extracted in very young children. Loss of anterior teeth in these children will have negative effect on the social well-being affecting the quality of life. Surgical extraction has the potency to induce a certain amount of dental fear and anxiety in young children. Thus, a conservative approach, although it is controversial, could be adopted and attempts are made to save root-fractured primary teeth, with extraction as the last choice [6, 7].

The surgical extrusion is considered a viable alternative for the management of crown-root fracture, when the fractures extend subgingivally and periodontal surgery is not recommended owing to esthetic reason. When compared with orthodontic extrusion, which involves placing either post or brackets in the exposed tooth surface, this treatment option allows the detection of additional fractures at the root. Moreover, studies show that surgical extrusion has an acceptable prognosis; approximately 80% of teeth treated are still in working condition after five years [3, 4]. It is comparatively easier to get the patients' cooperation since it is a shorter-duration procedure, less expensive, and the tooth can be in function for a longer duration [1–4].

In the present case report, the traumatised teeth were surgically extruded followed by the extraction of mobile tooth fragment in the coronal portion, splinted and stabilised. In the next appointment, the teeth were treated with pulpectomy and coronal restoration. On eight-week follow-up, the tooth exhibited no clinical signs of failure, such as mobility, tenderness, or pain. The outcome was successful in this case at one year, as there was no underlying pathology in the follow-up period suggesting no risk for the underlying permanent tooth germ. The long-term success of surgical extrusion depends on the cooperation of the child, the condition of the periodontal ligament, the vitality of the teeth, and the time lapsed following trauma. Thus, the conservative approach in the management of crown-root fracture in primary dentition should be emphasised. Further more studies of similar case reports are desirable in the management of crown-root fractures in primary dentition, before any recommendations to be made in the guidelines of the management of trauma.

In conclusion, this case report confirms that a multidisciplinary approach surgical extrusion is one of the alternative methods to manage the complicated crown-root fracture in a primary dentition.
